# Supplementary material for: Non-monotonic pressure dependence of the thermal conductivity of boron arsenide
Source: Nat Commun. 2019 Feb 19;10:827. doi: 10.1038/s41467-019-08713-0 (PMC6381145; doi:10.1038/s41467-019-08713-0)
Supplement: Supplementary file 1 — Supplementary Information [file 41467_2019_8713_MOESM1_ESM.pdf]

Non-monotonic Pressure Dependence of the Thermal Conductivity of Boron Arsenide
Supplementary Information

Navaneetha K. Ravichandran* and David Broido
Department of Physics, Boston College, Chestnut Hill, MA 02467, USA

* navaneeth.ravichandran@bc.edu

Supplementary Note 1. Pressure-dependent properties of cBN and MgO

In this section, we present additional pressure-dependent calculations on cubic Boron Nitride (cBN) and Magnesium Oxide (MgO). Supplementary Figure 1 (a) shows the evolution of the phonon dispersions in cBN with pressure. Both acoustic and optic modes increase in frequency due to the application of hydrostatic pressure, similar to MgO (shown in fig. 1 of the main text). We also present the change in frequency of the transverse optic (TO) mode at the Γ-point as the lattice is compressed due to hydrostatic pressure, and the pressure-volume curve at 300 K for cBN in the Supplementary Figures 1 (b) and (c) respectively. Our calculations are in good agreement with experiments in literature [1–3]. The curve in Supplementary Figure 1 (c) was rigidly shifted to match the experimental volume at zero pressure *only*, and the corresponding volume was used as V_0 in Supplementary Figure 1 (b) ($\sim$ 0.8% change in the lattice constant, only for these two plots).

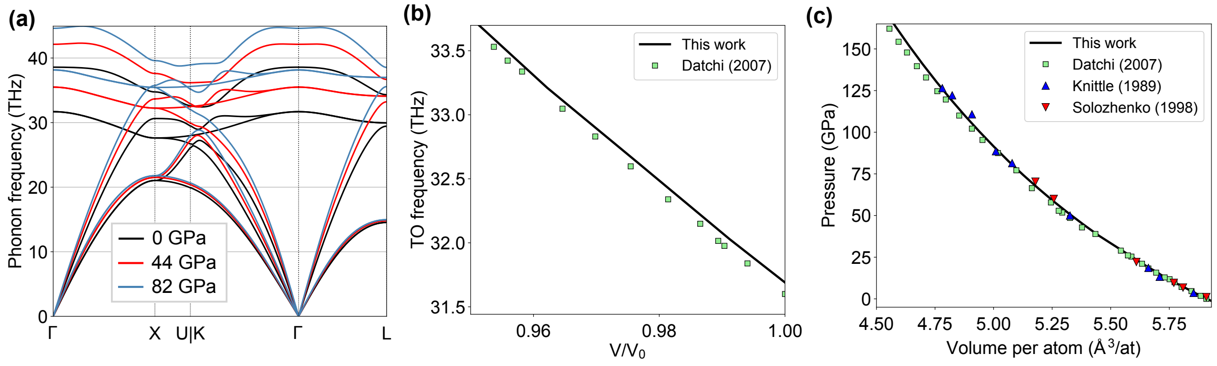

Supplementary Figure 1. Pressure-dependent properties of cBN. (a) Room temperature phonon dispersions of cBN at 0 GPa (black), 44 GPa (red) and 82 GPa (blue). (b) Evolution of the TO mode frequency at the Γ point as a function of volume at 300 K, compared with experiments from Datchi et al. [1]. The x-axis is scaled by the volume at zero pressure, V_0. (c) Calculated pressure-volume curve at 300 K compared with experiments from Datchi et al. [1], Knittle et al. [2] and Solozhenkho et al. [3].

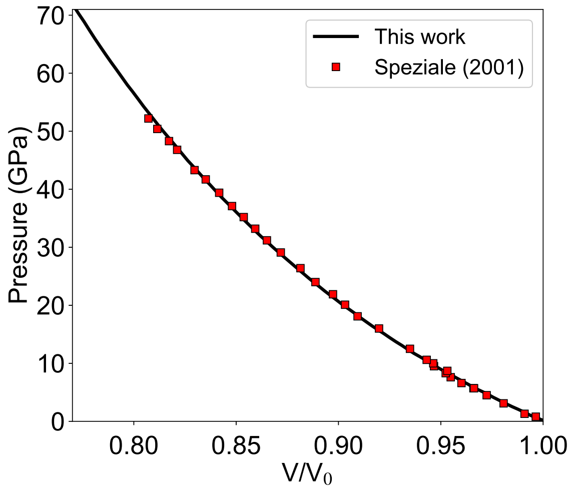

Supplementary Figure 2. Pressure-volume curve for MgO. Calculated pressure-volume curve for MgO at 300 K compared with experiments from Speziale et al. [4]. The x-axis is scaled by the volume at zero pressure, V_0.

For MgO, we present our calculations for the pressure-volume curve at 300 K in Supplementary Figure 2, and we get good agreement with the experiments in Ref. [4]. The curve in Supplementary Figure 2 was rigidly shifted to match the experimental volume at zero pressure *only* ($\sim$ 1.2% change in the lattice constant, only for this plot).

Supplementary Figure 3 (a) shows our calculated total three-phonon and process-wise four-phonon scattering rates of cBN at 300 K and at different pressures. There are two stark differences in the pressure dependence of the scattering rates between cBN and BAs (presented in the main text). First, the three-phonon scattering rates of heat carrying phonons (in the frequency range of 5-25 THz, see Supplementary Figure 3 (b)) are an order of magnitude stronger than the four-phonon scattering rates. Hence the effect of four-phonon scattering in cBN is weak at 300 K. Second, the three-phonon scattering rates decrease with increasing pressure. Similar trends for the pressure dependence of the three-phonon and four-phonon scattering rates are also observed for MgO (shown in Supplementary Figure 4). Since the three-phonon scattering rates, which dominate the total scattering rates, decrease with pressure, both the three-phonon limited thermal conductivity, $\kappa^{(3)}$ and the 3+4-phonon limited thermal conductivity, $\kappa^{(3+4)}$ of cBN and MgO increase with pressure unlike BAs, as shown in fig. 1 (b) and (c) of the main manuscript respectively.

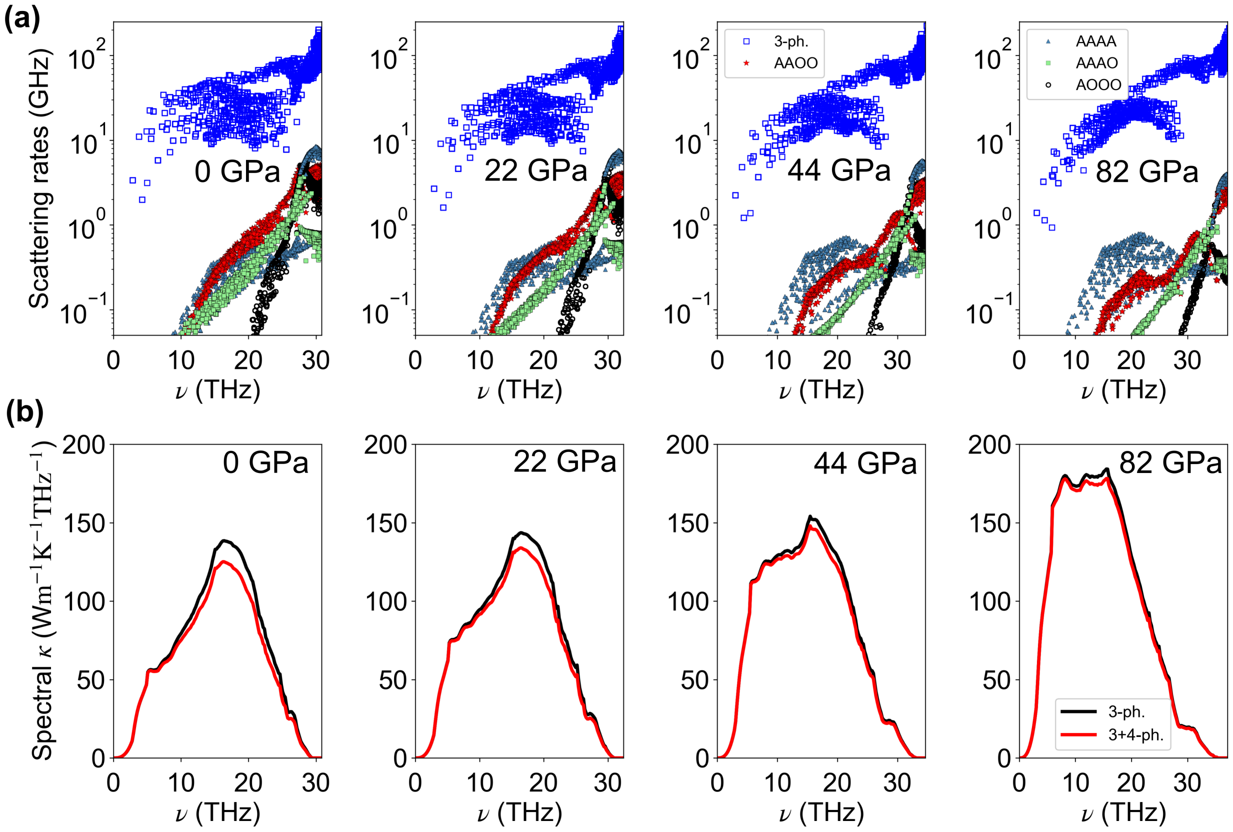

Supplementary Figure 3. Pressure-dependent scattering rates and spectral κ for cBN. (a) Calculated total three-phonon and process-wise four-phonon scattering rates of the acoustic modes of cBN at 300 K and at different pressures. (b) Calculated spectral contributions to $\kappa^{(3)}$ and $\kappa^{(3+4)}$ of the acoustic modes of cBN at 300 K and at different pressures. The spectral contributions to both $\kappa^{(3)}$ and $\kappa^{(3+4)}$ increase with pressure, and the percentage reduction in κ due to four-phonon scattering decreases with increasing pressure.

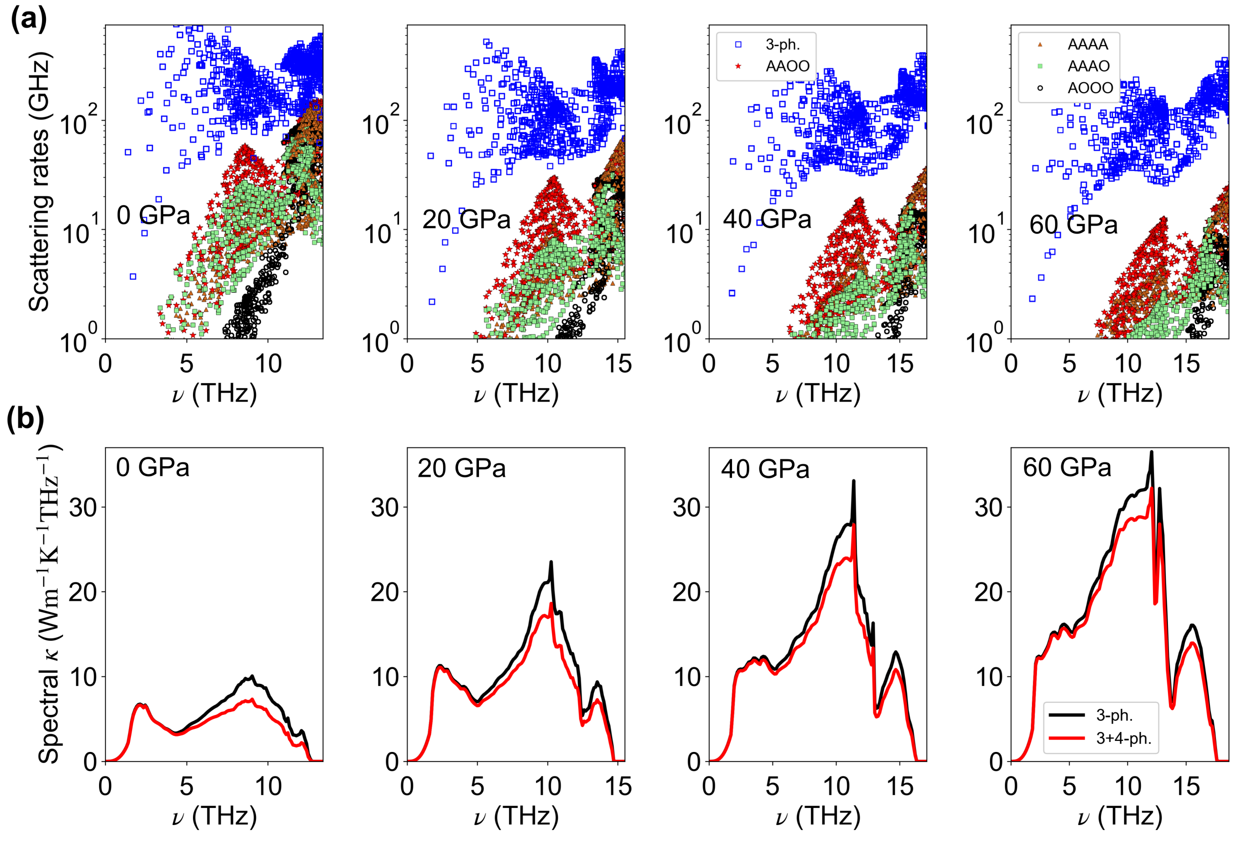

Supplementary Figure 4. Pressure-dependent scattering rates and spectral κ for MgO. (a) Calculated total three-phonon and process-wise four-phonon scattering rates of the acoustic modes of MgO at 300 K and at different pressures. (b) Calculated spectral contributions to $\kappa^{(3)}$ and $\kappa^{(3+4)}$ of the acoustic modes of MgO at 300 K and at different pressures. Both scattering rates and spectral κ have similar responses to increasing pressure as in cBN.

Supplementary Note 2. Pressure dependence of the thermal conductivity of natural BAs

In this section, we present $\kappa^{(3)}$ and $\kappa^{(3+4)}$ for BAs with naturally occurring isotopic composition of the constituent atoms (19.9% ^{10}B, 80.1% ^{11}B; As is isotopically pure). Supplementary Figures 5 (a) and (b) show that the pressure dependencies of $\kappa^{(3)}$ and $\kappa^{(3+4)}$ for natural BAs are qualitatively similar to the isotopically pure BAs results presented in the main text. Furthermore, Supplementary Figure 5 (c) shows that the pressure at which $\kappa^{(3+4)}$ of natural BAs peaks also shows a similar temperature dependence as the isotopically pure BAs.

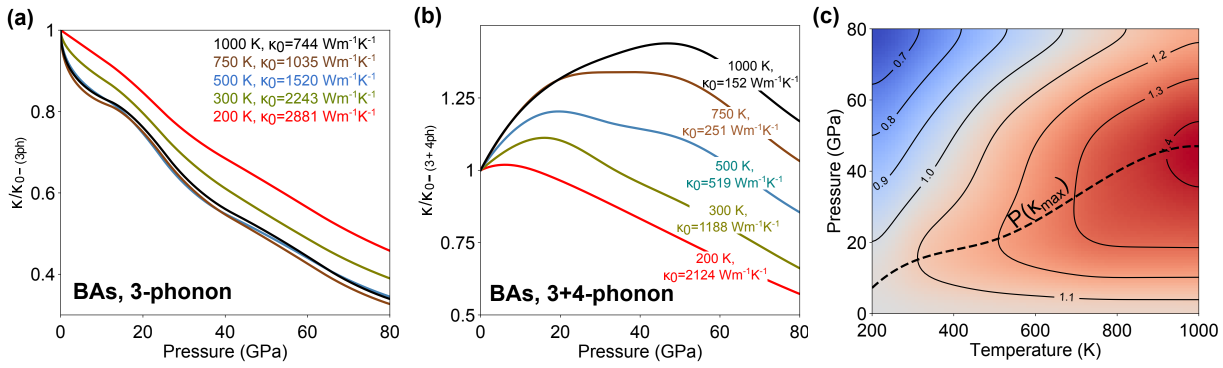

Supplementary Figure 5. Pressure-dependent thermal conductivity of natural BAs. (a) $\kappa^{(3)}$ of natural BAs versus pressure at different temperatures. (b) $\kappa^{(3+4)}$ of natural BAs versus pressure at different temperatures. All κ curves are scaled by the corresponding zero-pressure values listed within the figures. (c) 2D Colormap of $\kappa^{(3+4)}$ scaled by the corresponding zero-pressure value ($\kappa_0^{(3+4)}$) versus temperature and pressure along with the iso-$\kappa^{(3+4)}/\kappa_0^{(3+4)}$ lines, showing the non-monotonic behavior of $\kappa^{(3+4)}$ of natural BAs with pressure at each temperature. Also shown is the shifting position of the $\kappa^{(3+4)}$ peak on the pressure-temperature surface.

Supplementary Note 3. Pressure-dependent properties of BAs using PBEsol

In this section, we present the pressure-dependent $\kappa^{(3)}$ and $\kappa^{(3+4)}$ for isotopically pure BAs using ultrasoft pseudopotentials with PBEsol exchange-correlation functionals (generalized gradient approximation - GGA type) from the GBRV pseudopotential library [5]. For these calculations, kinetic energy cutoffs of 50 Ry for the wavefunctions and 200 Ry for the charge density, and a 8^3-shifted electronic **k**-grid produced a convergence of 6×10^{-4} Ry per unit cell for the total energy and 0.15 kbar per unit cell for the total stresses. For the force-displacement calculations, 5X5X5 supercells were used, for which a Γ-shifted electronic **k**-grid produced a convergence of less than 10^{-5} Ry/au for the forces. For the thermal conductivity calculations, the converged parameters were: 9^3 **q**-grid for the initial density functional perturbation theory calculation to obtain the bare harmonic interatomic force constants (IFCs) and 200 snapshots for the anharmonic IFCs for each point on the pressure-temperature grid, 17^3 **q**-grid for the solution of the Peierls-Boltzmann equation to obtain $\kappa^{(3)}$ and $\kappa^{(3+4)}$ using 11 nearest neighbors for the harmonic IFCs, 7 nearest neighbors for the cubic IFCs and 3 nearest neighbors for the quartic IFCs.

Supplementary Figure 6 (a) shows that the frequencies of the optic and the longitudinal acoustic (LA) modes of BAs, calculated with the PBEsol pseudopotentials, increase with pressure while the lower transverse acoustic (TA) branch undergoes weak softening, similar to results using the norm-conserving local density approximation (LDA) results presented in the main text. Supplementary Figure 6 (b) and (c) show that the monotonically decreasing $\kappa^{(3)}$ with pressure, the non-monotonic pressure dependence of $\kappa^{(3+4)}$ and the temperature-dependent shift in the pressure at which $\kappa^{(3+4)}$ peaks, are all observed in the calculated results with PBEsol, similar to the LDA calculations in the main text. Furthermore, the PBEsol results of the temperature-dependent shift in the peak-$\kappa^{(3+4)}$ pressure are shown in the pressure-temperature contour in Supplementary Figure 7 (a), and the opposing responses of the three-phonon and four-phonon scattering rates to pressure rise at 300 K and 750 K are shown in Supplementary Figure 7 (b). These features are also qualitatively similar to the LDA results presented in the main text.

Also, the percentage enhancement of $\kappa^{(3+4)}$ at its peak from the ambient pressure values are similar between LDA

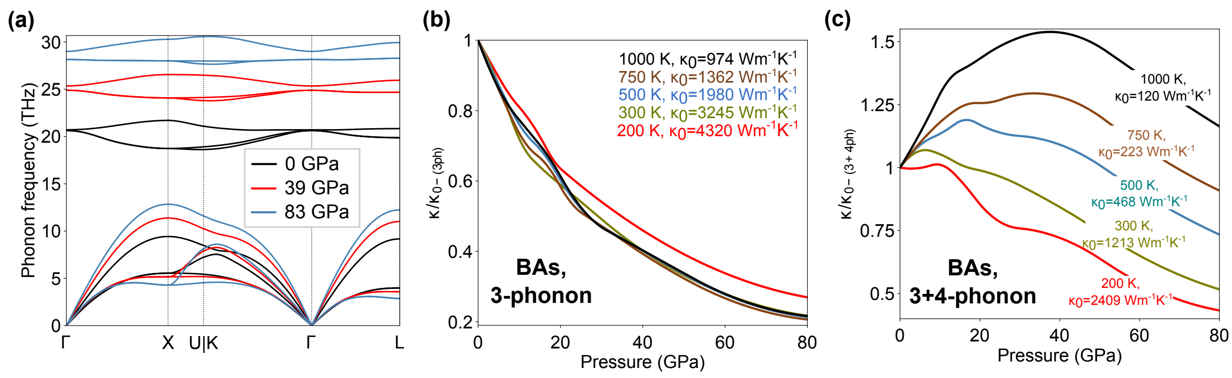

Supplementary Figure 6. Pressure-dependent properties of isotopically pure BAs with PBEsol. (a) Room temperature phonon dispersions of BAs at 0 GPa (black), 39 GPa (red) and 83 GPa (blue). (b) $\kappa^{(3)}$ of isotopically pure BAs versus pressure at different temperatures. (c) $\kappa^{(3+4)}$ of isotopically pure BAs versus pressure at different temperatures. All κ curves are scaled by the corresponding zero-pressure values listed within the figures.

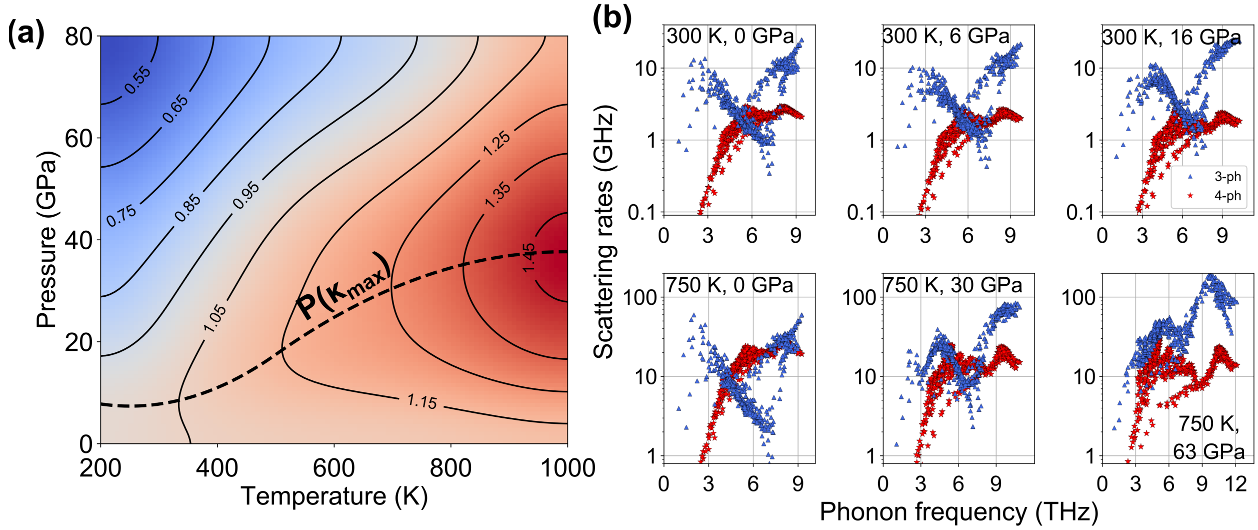

Supplementary Figure 7. Non-monotonic pressure dependence of $\kappa^{(3+4)}$ in isotopically pure BAs with PBEsol. (a) Pressure-temperature colormap of $\kappa^{(3+4)}$ of the isotopically pure BAs scaled by the corresponding zero-pressure value at each temperature ($\kappa_{L,0}^{(3+4)}$). Also shown is the shifting position of the thermal conductivity peak on the pressure-temperature surface. (b) Comparison of three-phonon and four-phonon scattering rates of the acoustic modes in BAs at 300 K and 750 K at different pressures. The center plots [(300 K, 6 GPa) and (750 K, 30 GPa)] are the conditions close the $\kappa^{(3+4)}$-peak in Supplementary Figure 6 (c) at the corresponding temperatures.

and PBEsol calculations (7% [PBEsol] vs. 11% [LDA] at 300 K; 53% [PBEsol] vs. 44% [LDA] at 1000 K). The pressure at which $\kappa^{(3+4)}$ peaks is similar between the LDA and PBEsol calculations at high temperatures (17 GPa [PBEsol] vs. 19 GPa [LDA] at 500 K; 30 GPa [PBEsol] vs. 33 GPa [LDA] at 750 K; 39 GPa [PBEsol] vs. 45 GPa [LDA] at 1000 K), while there is a slightly larger difference at room temperature (7 GPa [PBEsol] vs. 17.5 GPa [LDA]). Overall, slightly larger lattice constants, lower group velocities, smaller three-phonon scattering rates and larger four-phonon scattering rates using PBEsol [see Supplementary Figures 8 (b) and (c)] result in slightly larger $\kappa^{(3)}$ and smaller $\kappa^{(3+4)}$ compared to those for LDA. The temperature and pressure dependence of $\kappa^{(3)}$ and $\kappa^{(3+4)}$ for natural BAs also compare similarly between the LDA (Supplementary Figure 5) and the PBEsol calculations (Supplementary Figure 9).

Finally, the calculated $\kappa^{(3+4)}$ of isotopically pure BAs at ambient conditions using LDA and PBEsol agree to within $\sim$ 9% (1213 $Wm^{-1}K^{-1}$ [PBEsol] vs. 1331 $Wm^{-1}K^{-1}$ [LDA]) and those of natural BAs agree to within $\sim$ 10% (1077 $Wm^{-1}K^{-1}$ [PBEsol] vs. 1188 $Wm^{-1}K^{-1}$ [LDA]), which are also in good agreement with the cross-study

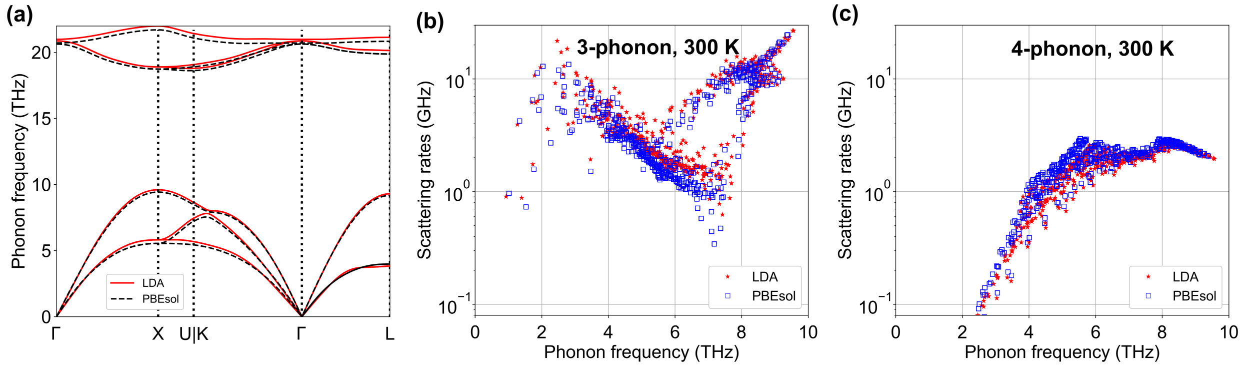

Supplementary Figure 8. Comparison between LDA and PBEsol calculations for BAs at ambient conditions. (a) Phonon dispersions of BAs. (b) Three-phonon scattering rates of the acoustic modes of BAs. (c) Four-phonon scattering rates of the acoustic modes of BAs. The phonon dispersions, three-phonon scattering rates and four-phonon scattering rates for BAs at ambient conditions are in good agreement between the LDA and PBEsol calculations.

average of 1140 ± 15% $\mathrm{Wm^{-1}K^{-1}}$ from the recent experiments on natural BAs [6–9]. Thus, the unique pressure and temperature-dependent features of the $\kappa^{(3+4)}$ of BAs are robust across the two pseudopotentials used in this study, and the observed small quantitative differences are consistent with that reported in the literature for the thermal and thermodynamic properties of BAs and other materials using these two exchange-correlation functionals [10–13].

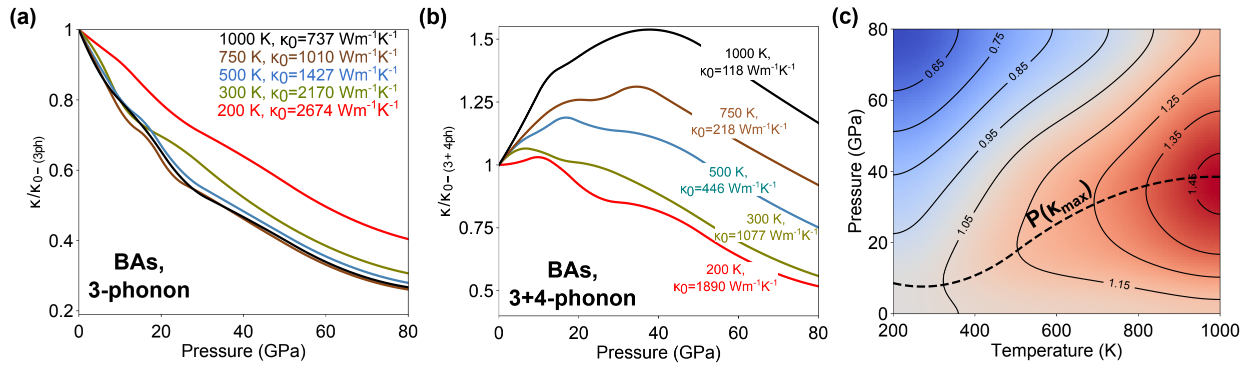

Supplementary Figure 9. Pressure dependence of the thermal conductivity of natural BAs with PBEsol. (a) $\kappa^{(3)}$ of BAs with naturally occurring isotopic mix versus pressure at different temperatures. (b) $\kappa^{(3+4)}$ of BAs with naturally occurring isotopic mix versus pressure at different temperatures. All κ curves are scaled by the corresponding zero-pressure values listed within the figures. (c) Pressure-temperature colormap of $\kappa^{(3+4)}$ of BAs with naturally occurring isotopic mix, scaled by the corresponding zero-pressure value at each temperature ($\kappa_{L,0}^{(3+4)}$).

Supplementary Note 4. Thermal conductivity of BAs without three-phonon scattering

Although four-phonon scattering strongly affects $\kappa^{(3+4)}$ of BAs even at room temperature, it is still significantly weaker than three-phonon scattering in most regions of the Brillouin zone. Only within the frequency range of ∼ 4-8 THz, the four-phonon scattering rates are comparable to the three-phonon scattering rates at ambient conditions (see figs. 3(a) and 4(b) in the main text), primarily because of the unusually weak three-phonon scattering in that frequency range, and not due to particularly strong four-phonon scattering in BAs. Note that away from the 4-8 THz region, the four-phonon scattering rates are much smaller than the corresponding three-phonon scattering rates. To further elucidate this important point, Supplementary Figure 10 shows the pressure dependence of three-phonon, four-phonon and 3+4-phonon limited thermal conductivity of BAs. Thermal conductivity including only four-phonon scattering ($\kappa^{(4)}$) in Supplementary Figure 10 (b) is much larger than $\kappa^{(3)}$ in Supplementary Figure 10 (a) and $\kappa^{(3+4)}$

in Supplementary Figure 10 (c) at room temperature, indicating that four-phonon scattering is still weak in BAs compared to other strongly anharmonic materials like sodium chloride [12] and lead telluride [14].

At temperatures higher than 500 K and at ambient pressure, four-phonon scattering rapidly strengthens and $\kappa^{(4)}$ becomes comparable to $\kappa^{(3)}$. For example, at 750 K and ambient pressure, $\kappa^{(3)}$ is 1271 $\mathrm{Wm^{-1}K^{-1}}$, while $\kappa^{(4)}$ is 1733 $\mathrm{Wm^{-1}K^{-1}}$. However, in this high temperature region, pressure rise strengthens the three-phonon scattering processes, causing $\kappa^{(3)}$ to decrease monotonically, but weakens the four-phonon processes, thus resulting in $\kappa^{(4)}[P > 0] > \kappa^{(4)}[P = 0]$ even beyond 70 GPa, as shown in Supplementary Figure 10 (b). Also, as described in the main text, pressure rise strengthens AAAA four-phonon scattering channels but weakens AAOO and AAAO four-phonon scattering channels in BAs. These opposing responses to pressure rise, even among different four-phonon scattering channels, cause a non-monotonic pressure dependence of $\kappa^{(4)}$ too, as shown in Supplementary Figure 10 (b). Note that the origin of the non-monotonic pressure dependence of $\kappa^{(4)}$ (Supplementary Figure 10 (b)) is different from that of $\kappa^{(3+4)}$ presented in the main text and in Supplementary Figure 10 (c), which is caused by the opposing responses of three-phonon and four-phonon scattering channels to pressure rise.

However, quantitative conclusions about the pressure dependence of $\kappa^{(3+4)}$ cannot be drawn just from the plots of $\kappa^{(3)}$ and $\kappa^{(4)}$ (Supplementary Figures 10 (a) and (b) respectively), since the competition between three-phonon and four-phonon processes occurs within a small frequency range ($\sim$ 4-8 THz), while $\kappa^{(3)}$ and $\kappa^{(4)}$ are averaged over the entire phonon spectrum. For example, at 200 K and ambient pressure, $\kappa^{(4)} > 10\ \kappa^{(3)}$; yet $\kappa^{(3+4)} \sim 0.5\ \kappa^{(3)}$ is still affected by four-phonon scattering. Thus, the full spectral information about the rates of different phonon scattering mechanisms and their pressure dependencies must be considered (as in fig. 3 of the main text) to predict the pressure dependence of $\kappa^{(3+4)}$ of BAs.

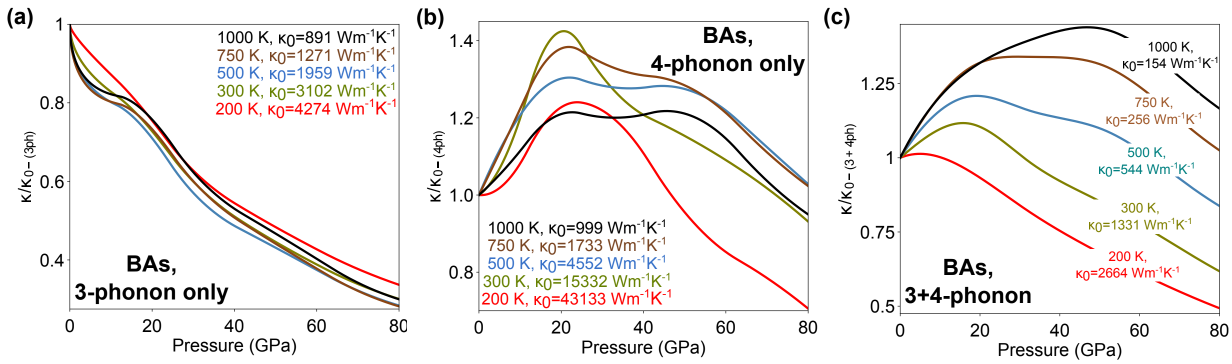

Supplementary Figure 10. Relative strengths of three-phonon and four-phonon scattering in BAs. Three-phonon only (a), four-phonon only (b) and 3+4-phonon (c) limited thermal conductivity of isotopically pure BAs. Figures (a) and (c) are the same as in the main text (fig. 2 (b) and (c) respectively). Thermal conductivity including only four-phonon scattering ($\kappa^{(4)}$) in (b) is much larger than $\kappa^{(3)}$ (a) and $\kappa^{(3+4)}$ (c) at room temperature, indicating that four-phonon scattering is still weak in BAs compared to other strongly anharmonic materials like sodium chloride [12] and lead telluride [14]. However, at temperatures higher than 500 K and at ambient pressure, four-phonon scattering rapidly strengthens and $\kappa^{(4)}$ becomes comparable to $\kappa^{(3)}$.

Supplementary Note 5. Pressure dependence of the scattering phase space in BAs

In this section, we present the pressure dependence of the normalized scattering phase space of various three-phonon and four-phonon scattering channels in BAs at different temperatures. The normalized three-phonon scattering phase space is defined as [13, 15]:

$$P_3 = \frac{2}{3N_p^3 V_{BZ}^2} \sum_{j,j',j''} \int \mathrm{d}\mathbf{q}^3 \mathrm{d}\mathbf{q}'^3 \Bigg(\delta\left[\omega_j(\mathbf{q}) + \omega_{j'}(\mathbf{q}') - \omega_{j''}(\mathbf{q}+\mathbf{q}'-\mathbf{G})\right] + \frac{1}{2}\delta\left[\omega_j(\mathbf{q}) - \omega_{j'}(\mathbf{q}') - \omega_{j''}(\mathbf{q}-\mathbf{q}'-\mathbf{G})\right] \Bigg) \tag{1}$$

where N_p is the number of phonon polarizations, V_{BZ} is the volume of the Brillouin zone, $\omega_j(\mathbf{q})$ is the frequency of the phonon mode $(\mathbf{q}, j)$ and $\mathbf{G}$ is a reciprocal lattice vector that brings $\mathbf{q} \pm \mathbf{q}'$ into the first Brillouin zone. Similarly the normalized four-phonon phase space can be defined as:

$$P_4 = \frac{6}{7N_p^4 V_{BZ}^3} \sum_{j,j',j'',j'''} \int \mathrm{d}\mathbf{q}^3 \mathrm{d}\mathbf{q}'^3 \mathrm{d}\mathbf{q}''^3 \Bigg(\frac{1}{6}\delta\left[\omega_j(\mathbf{q}) - \omega_{j'}(\mathbf{q}') - \omega_{j''}(\mathbf{q}'') - \omega_{j'''}(\mathbf{q} - \mathbf{q}' - \mathbf{q}'' - \mathbf{G})\right] + \frac{1}{2}\delta\left[\omega_j(\mathbf{q}) + \omega_{j'}(\mathbf{q}') - \omega_{j''}(\mathbf{q}'') - \omega_{j'''}(\mathbf{q} + \mathbf{q}' - \mathbf{q}'' - \mathbf{G})\right] + \frac{1}{2}\delta\left[\omega_j(\mathbf{q}) + \omega_{j'}(\mathbf{q}') + \omega_{j''}(\mathbf{q}'') - \omega_{j'''}(\mathbf{q} + \mathbf{q}' + \mathbf{q}'' - \mathbf{G})\right] \Bigg) \quad (2)$$

The three-phonon (P_3) and four-phonon (P_4) phase spaces are normalized such that the energy-unrestricted phase space, where all δ-functions are set to unity, is equal to one second for both P_3 and P_4. Supplementary Figures 11 (a) and (b) show the three-phonon and four-phonon scattering phase spaces respectively as functions of pressure at different temperatures, broken down by individual scattering channels for the acoustic modes of BAs. The phase spaces of all three-phonon scattering channels increase with pressure, while those of four-phonon scattering channels decrease with pressure. Furthermore, the AAA scattering channel has the largest three-phonon phase space, and the AAOO scattering channel has the largest four-phonon phase space. The AOOO four-phonon scattering channel has identically zero phase space for BAs at all temperatures and pressures, since the δ-functions cannot be satisfied for this combination of phonons; thus it is not shown in Supplementary Figure 11 (b).

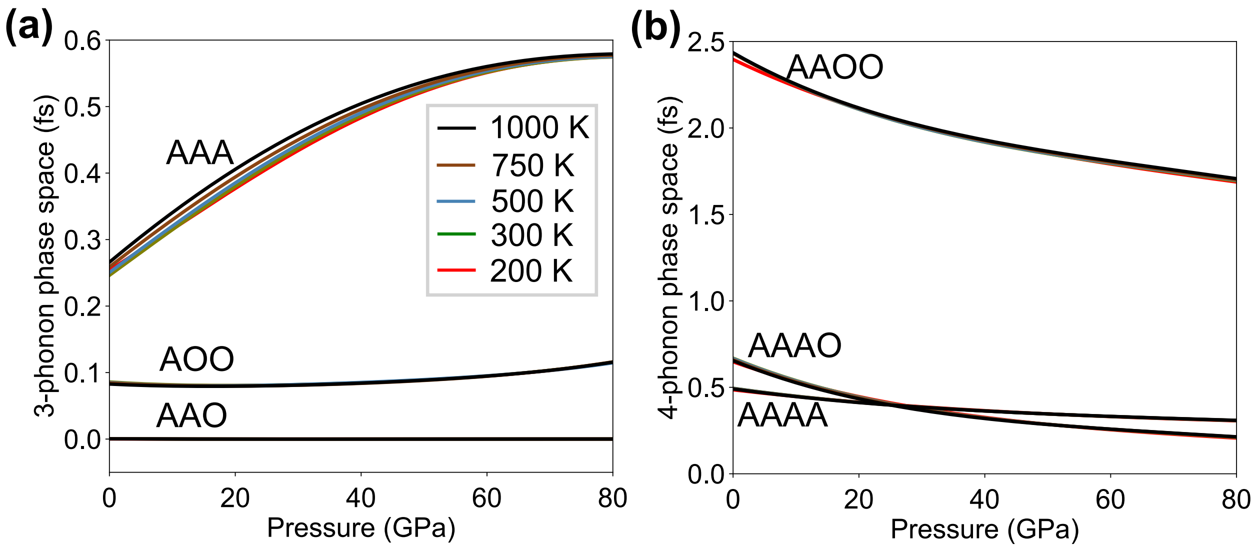

Supplementary Figure 11. Pressure-dependent scattering phase space of BAs. Three-phonon (a) and four-phonon (b) scattering phase space of the acoustic modes of BAs calculated from Supplementary Equations 1 and 2 respectively for different scattering channels at different temperatures and pressures. The AOOO four-phonon scattering channel has zero phase space for BAs at all conditions, thus not shown in (b). The phase spaces of three-phonon channels increase with pressure, while those of four-phonon channels decrease with pressure.

Quantitative comparisons between P_3 and P_4 alone cannot be used to draw conclusions about the pressure-dependence of $\kappa^{(3+4)}$ of BAs, since the corresponding scattering rates, which eventually determine $\kappa^{(3+4)}$, also contain additional factors (Bose factors and the anharmonic scattering matrix elements), that (i) can have complex pressure dependencies different from those of the δ-functions in the phase space, and (ii) are, in fact, orders of magnitude different for three-phonon and four-phonon scattering channels. Furthermore, P_3 and P_4 are averaged over the entire phonon spectrum (Supplementary Equations 1 and 2), while the competition between three-phonon and four-phonon processes occurs only within a small frequency range for the acoustic modes of BAs, as described in the main text. Thus, as mentioned in the main text, the phase space - being a harmonic quantity, can only present a part of the overall

picture describing the pressure-driven competition between the three-phonon and four-phonon scattering strengths in BAs.

Supplementary Note 6. Temperature dependence of phonon scattering rates in BAs

In this section, we describe the temperature dependence of three-phonon and four-phonon scattering rates in BAs. Supplementary Figure 12 (a) and (b) show that four-phonon scattering rates have stronger temperature dependence than three-phonon processes at 0 GPa and 50 GPa respectively, consistent with the previous findings at ambient pressure [16]. This stronger temperature dependence of four-phonon scattering is the root cause of the temperature dependence of the non-monotonic behavior of $\kappa^{(3+4)}$ with pressure rise, as observed in the main text. For example, at both 0 GPa and 50 GPa, four-phonon scattering increases $\sim$ ten-fold when the temperature increases from room temperature to 1000 K, while three-phonon scattering only undergoes a two to four-fold enhancement (Supplementary Figure 12). Thus, the pressure-driven competition between three-phonon and four-phonon scattering channels becomes strongly temperature-dependent, as described in the main text.

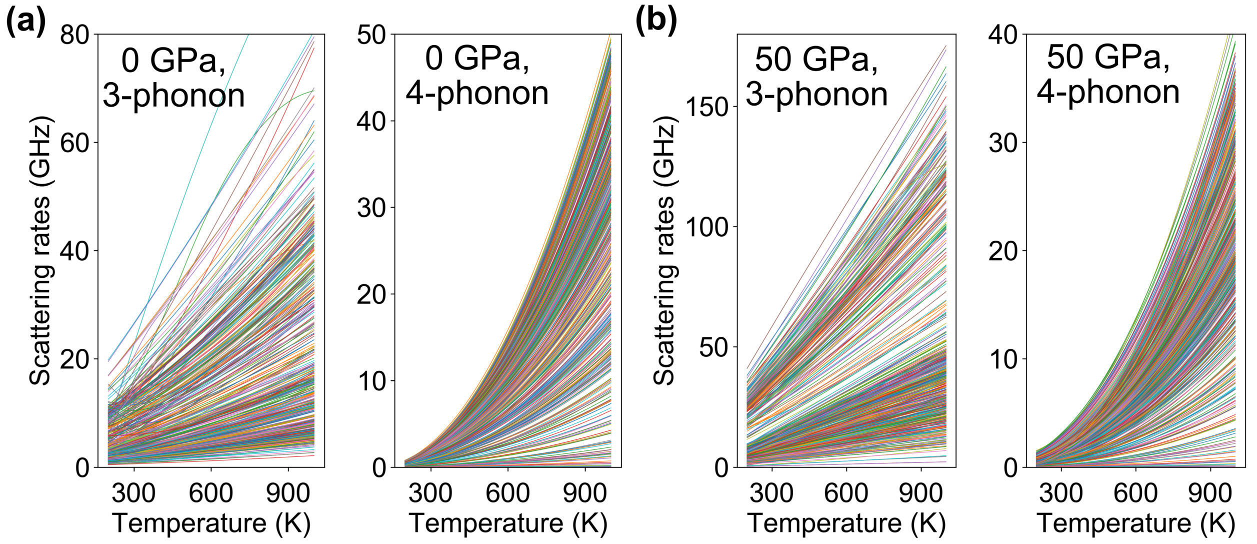

Supplementary Figure 12. Temperature dependence of three-phonon and four-phonon scattering rates of BAs. Three-phonon and four-phonon scattering rates of different acoustic phonon modes in the Brillouin zone as functions of temperature at (a) 0 GPa and (b) 50 GPa. Each color represents a separate phonon mode. Three-phonon scattering rates show weaker temperature dependence at both low and high pressures compared to four-phonon scattering rates, consistent with previous findings at ambient pressure [16].

Supplementary Note 7. Pressure dependence of the thermal conductivity of BSb

In this section, we present additional results for the pressure-dependent properties of Boron Antimonide (BSb). Supplementary Figure 13 (a) shows that the frequencies of the optic and the LA modes of BSb increase with pressure while the lower TA branch undergoes weak softening, similar to BAs. For the isotopically pure BSb, $\kappa^{(3)}$ decreases with pressure and $\kappa^{(3+4)}$ shows a non-monotonic pressure dependence, as shown in Supplementary Figures 13 (b) and (c) respectively, also similar to BAs.

On the other hand, BSb with naturally occurring isotopic composition of the constituent atoms (19.9% ^{10}B, 80.1% ^{11}B, 57.2% ^{121}Sb and 42.79% ^{123}Sb) shows a qualitatively different pressure dependence of $\kappa^{(3)}$ compared to BAs and isotopically pure BSb, as shown in Supplementary Figure 14 (a). Below 500 K, even $\kappa^{(3)}$ of natural BSb shows a non-monotonic pressure dependence, which disappears above 500 K. This non-monotonic behavior at low temperature is also carried over to $\kappa^{(3+4)}$, as shown in Supplementary Figure 14 (b). Above 500 K, $\kappa^{(3+4)}$ shows a similar non-monotonic behavior as BAs and isotopically pure BSb, with the percentage enhancement of peak $\kappa^{(3+4)}$ from

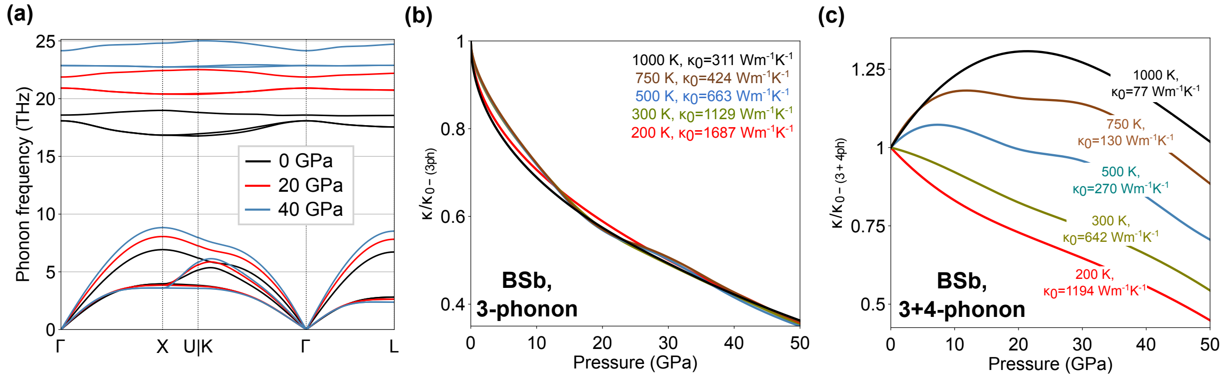

Supplementary Figure 13. Pressure-dependent phonons and thermal conductivity of isotopically pure BSb. (a) Room temperature phonon dispersions of BSb at 0 GPa (black), 20 GPa (red) and 40 GPa (blue). (b) $\kappa^{(3)}$ of isotopically pure BSb versus pressure at different temperatures. (c) $\kappa^{(3+4)}$ of isotopically pure BSb versus pressure at different temperatures. All κ curves are scaled by the corresponding zero-pressure values listed within the figures.

the zero pressure value increasing with increasing temperature. The presence of a peak $\kappa^{(3)}$ at low temperature also results in the peak pressure position of $\kappa^{(3+4)}$ decreasing at first, then increasing with increasing temperature, as shown in Supplementary Figure 14 (c).

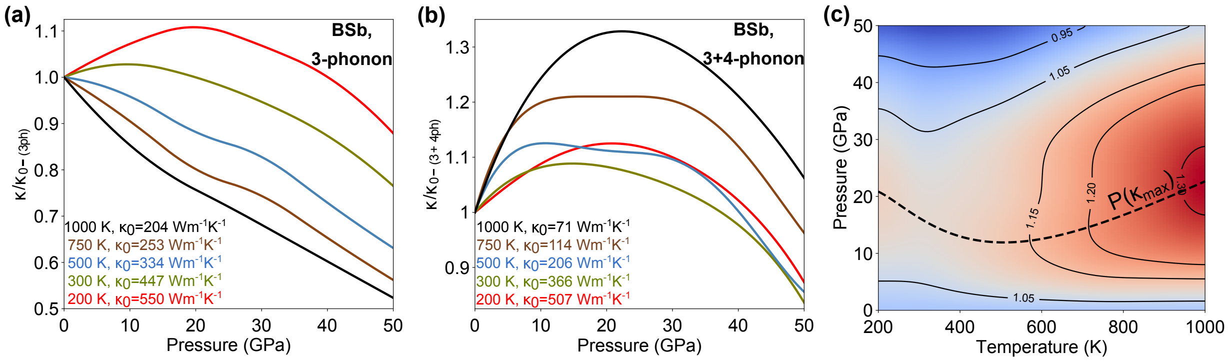

Supplementary Figure 14. Pressure-dependent thermal conductivity of natural BSb. (a) $\kappa^{(3)}$ of natural BSb versus pressure at different temperatures. (b) $\kappa^{(3+4)}$ of natural BSb versus pressure at different temperatures. All κ curves are scaled by the corresponding zero-pressure values listed within the figures. (c) 2D Colormap of $\kappa^{(3+4)}$ scaled by the corresponding zero-pressure value ($\kappa_0^{(3+4)}$) versus temperature and pressure along with the iso-$\kappa^{(3+4)}/\kappa_0^{(3+4)}$ lines, showing the non-monotonic behavior of $\kappa^{(3+4)}$ of natural BSb with pressure at each temperature. Also shown is the shifting position of the peak $\kappa^{(3+4)}$ on the pressure-temperature surface.

The principal reason for the non-monotonic pressure dependence of $\kappa^{(3)}$ at low temperature is the strong phonon-isotope scattering in natural BSb. Similar to BAs, the largest contribution to $\kappa^{(3+4)}$ of isotopically pure BSb comes from the frequency range where three-phonon scattering is the weakest (4-6 THz). Supplementary Figure 15 shows that, in this frequency range, phonon-isotope scattering rates are stronger than the three-phonon and four-phonon scattering rates in BSb at 200 K. Furthermore, the phonon-isotope scattering rates show weak reduction with increasing pressure at 200 K. Thus, the total phonon scattering rates, including three-phonon, four-phonon and phonon-isotope scattering, initially decrease with increase in pressure in the 4-6 THz frequency range at 200 K and between 0-20 GPa. This reduction in total scattering rates, along with the increasing group velocities of the LA mode and one of the TA modes with pressure, increases $\kappa^{(3+4)}$ of natural BSb at 200 K from 0-20 GPa.

As the pressure increases beyond 20 GPa, Supplementary Figure 15 shows that the three-phonon scattering rates increase and dominate over the phonon-isotope scattering rates, due to the weakening of the bunching effect described

in the main text for BAs. Thus, $\kappa^{(3+4)}$ of natural BSb at high pressure is dominated by three-phonon scattering, resulting in a decreasing $\kappa^{(3+4)}$ with increasing pressure beyond 20 GPa at 200 K, similar to the isotopically pure BSb.

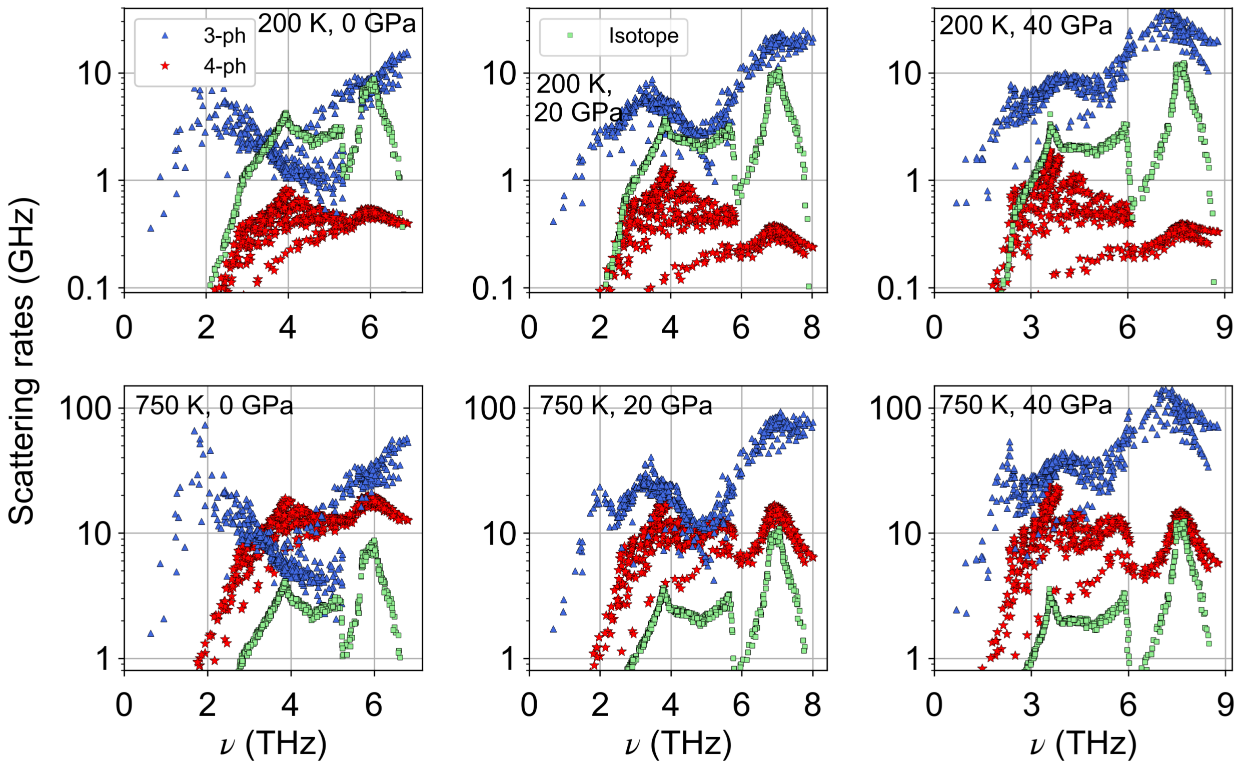

Supplementary Figure 15. Effect of isotope scattering on the thermal conductivity of natural BSb. Three-phonon (blue triangles), four-phonon (red stars) and phonon-isotope scattering rates (green squares) at 200 K (top three panels) and 750 K (bottom three panels) from zero to 40 GPa. From zero to $\lesssim$20 GPa, phonon-isotope scattering dominates total scattering rates at 200 K in the 4-6 THz frequency range, where the largest contribution to isotopically pure $\kappa^{(3+4)}$ comes from. Thus, the opposing responses of three-phonon and four-phonon scattering rates to pressure is not important at 200 K between 0 to $\sim$20 GPa, and so both natural $\kappa^{(3)}$ and natural $\kappa^{(3+4)}$ increase with pressure, similar to MgO. However, the pressure dependence of natural $\kappa^{(3)}$ and natural $\kappa^{(3+4)}$ is similar to that of isotopically pure BSb and BAs at 200 K beyond $\sim$20 GPa and at 750 K for all pressures, since the isotopic scattering rates are weaker than 3+4-phonon scattering rates under these conditions.

At temperatures higher than 500 K, both three-phonon and four-phonon scattering rates are stronger than the temperature-independent phonon-isotope scattering (see Supplementary Figure 15 for scattering rates at 750 K). Therefore, the pressure-dependent behaviors of both $\kappa^{(3)}$ and $\kappa^{(3+4)}$ for natural BSb are similar to the isotopically pure BSb, as shown in Supplementary Figure 14. It is worth noting that, while the isotopic compositions of Boron in both natural BAs and natural BSb are the same, Antimony has a large isotopic composition while Arsenic is isotopically pure. Hence, the phonon-isotope scattering is significantly stronger in natural BSb and reduces $\kappa^{(3+4)}$ by 58% from the isotopically pure value at 200 K, while the corresponding reduction is only 20% in natural BAs.

Supplementary Note 8. Anharmonic phonon renormalization in BAs and BSb

In this section, we describe the effect of anharmonic phonon renormalization on the phonon and thermal transport properties of BAs and BSb. The renormalization procedure used in this work has been described in our prior work [12], and includes the effects of anharmonicity at finite temperature and the zero-point motion of atoms. Supplementary Figure 16 (a) and (b) show that the effect of renormalization is small on the phonon dispersions of BAs at ambient pressure and 75 GPa at 300 K. From Supplementary Figure 16 (c) we find that, for BAs, the renormalization procedure

causes $\lesssim$ 12% difference in $\kappa^{(3)}$ at ambient pressure throughout the temperature range considered, while the difference in $\kappa^{(3+4)}$ is $\lesssim$ 8%. For BSb, the renormalization procedure causes $\lesssim$ 15% difference in $\kappa^{(3)}$, while the difference in $\kappa^{(3+4)}$ is $\lesssim$ 9%. Thus, the effect of phonon renormalization is weak in BAs and BSb, as expected for high κ, weakly-anharmonic materials.

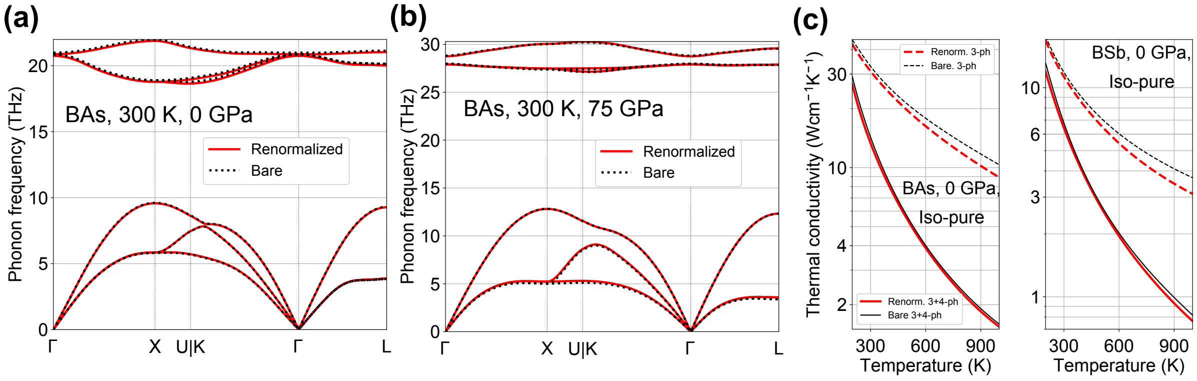

Supplementary Figure 16. Anharmonic phonon renormalization in BAs and BSb. Comparison of phonon dispersions of BAs at 300 K and pressures of (a) 0 GPa and (b) 75 GPa with and without renormalization. (c) Comparison of $\kappa^{(3)}$ and $\kappa^{(3+4)}$ for BAs and BSb as functions of temperature at ambient pressure, with and without renormalization.

Supplementary Note 9. Phonon renormalization and point defect scattering in MgO

In this section, we describe the effect of anharmonic phonon renormalization and point defect scattering on $\kappa^{(3+4)}$ of MgO. We find from Supplementary Figure 17 (a) that the effect of phonon renormalization is weak in MgO and cannot explain the discrepancy with experiments from Ref. [17]. The observed discrepancy could be attributed to the presence of impurities in the sample used in Ref. [17]. For example, addition of Fe impurities (500 parts per million [ppm] as mass defects on the Mg site) to our calculation improves the qualitative and quantitative agreement with the experiments, as shown in Supplementary Figure 17 (b). Addition of Fe impurities lowers the $\kappa^{(3+4)}$ of natural MgO by $\sim$ 9.5% at 0 GPa and $\sim$ 15% at 60 GPa. Pressure-dependent weakening of three-phonon and four-phonon scattering rates (Supplementary Figure 4 (a)), and the pressure-driven increase in phonon group velocities cause a larger absolute effect of Fe impurity scattering on $\kappa^{(3+4)}$ of natural MgO at 60 GPa compared to that at 0 GPa in Supplementary Figure 17 (b).

Supplementary Note 10. Phonon scattering rates and anharmonic free energy

In this section, we present the expressions for various phonon scattering probabilities that appear in the Peierls-Boltzmann equation (PBE) for phonon transport (equation 3 of the main manuscript) and the expression for the fourth-order anharmonic free energy used to determine the pressure. The three-phonon scattering probabilities ($W^{(\pm)}_{\lambda\lambda_1\lambda_2}$) are given by,

$$\begin{aligned} W^{(+)}_{\lambda\lambda_1\lambda_2} &= \frac{2\pi}{\hbar^2}\left|\Psi_{\lambda\lambda_1(-\lambda_2)}\right|^2\left(n^0_{\lambda_1}-n^0_{\lambda_2}\right)\delta\left(\omega_\lambda+\omega_{\lambda_1}-\omega_{\lambda_2}\right) \\ W^{(-)}_{\lambda\lambda_1\lambda_2} &= \frac{2\pi}{\hbar^2}\left|\Psi_{\lambda(-\lambda_1)(-\lambda_2)}\right|^2\left(1+n^0_{\lambda_1}+n^0_{\lambda_2}\right)\delta\left(\omega_\lambda-\omega_{\lambda_1}-\omega_{\lambda_2}\right) \end{aligned} \quad (3)$$

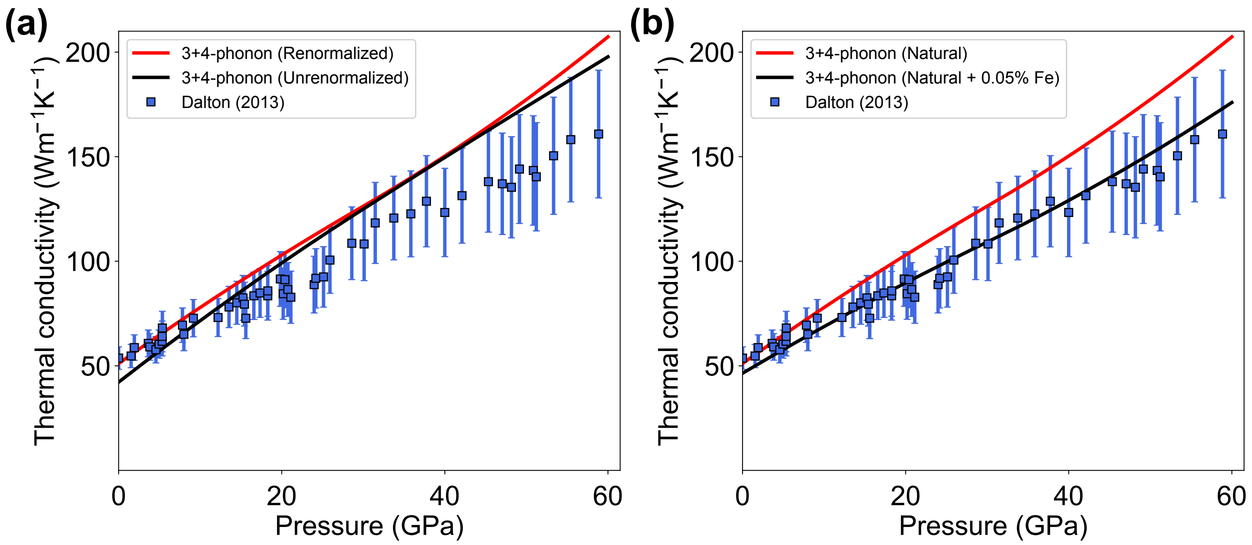

Supplementary Figure 17. Phonon renormalization and point defect scattering in MgO. (a) $\kappa^{(3+4)}$ of natural MgO with and without phonon renormalization as a function of pressure, compared with experiments [17]. (b) Renormalized $\kappa^{(3+4)}$ of natural MgO, and natural MgO with 500 ppm Fe impurities on the Mg site as functions of pressure, compared with experiments [17]

and the four-phonon scattering probabilities ($Y^{(1,2,3)}_{\lambda\lambda_1\lambda_2\lambda_3}$) are given by,

$$\begin{aligned}
Y^{(1)}_{\lambda\lambda_1\lambda_2\lambda_3} &= \frac{2\pi}{\hbar^2}\left|\Psi_{\lambda(-\lambda_1)(-\lambda_2)(-\lambda_3)}\right|^2 \frac{n^0_{\lambda_1} n^0_{\lambda_2} n^0_{\lambda_3}}{n^0_\lambda}\delta\left(\omega_\lambda - \omega_{\lambda_1} - \omega_{\lambda_2} - \omega_{\lambda_3}\right) \\
Y^{(2)}_{\lambda\lambda_1\lambda_2\lambda_3} &= \frac{2\pi}{\hbar^2}\left|\Psi_{\lambda\lambda_1(-\lambda_2)(-\lambda_3)}\right|^2 \frac{\left(1+n^0_{\lambda_1}\right) n^0_{\lambda_2} n^0_{\lambda_3}}{n^0_\lambda}\delta\left(\omega_\lambda + \omega_{\lambda_1} - \omega_{\lambda_2} - \omega_{\lambda_3}\right) \\
Y^{(3)}_{\lambda\lambda_1\lambda_2\lambda_3} &= \frac{2\pi}{\hbar^2}\left|\Psi_{\lambda\lambda_1\lambda_2(-\lambda_3)}\right|^2 \frac{\left(1+n^0_{\lambda_1}\right)\left(1+n^0_{\lambda_2}\right) n^0_{\lambda_3}}{n^0_\lambda}\delta\left(\omega_\lambda + \omega_{\lambda_1} + \omega_{\lambda_2} - \omega_{\lambda_3}\right)
\end{aligned} \tag{4}$$

where $-\lambda$ represents a phonon mode $((-\mathbf{q})\,s)$ when λ represents a phonon mode $(\mathbf{q}s)$ with wavevector $\mathbf{q}$ and polarization s, and $\Psi_{\lambda\lambda_1\lambda_2} \equiv \Psi_{\mathbf{q}s\mathbf{q}'s'\mathbf{q}''s''}$ and $\Psi_{\lambda\lambda_1\lambda_2\lambda_3} \equiv \Psi_{\mathbf{q}s\mathbf{q}'s'\mathbf{q}''s''\mathbf{q}'''s'''}$ are the third-order and fourth-order matrix elements given by:

$$\begin{aligned}
\Psi_{\lambda\lambda_1\lambda_2} &= \Psi_{\mathbf{q}s,\mathbf{q}_1s_1,\mathbf{q}_2s_2} \\
&= (\hbar/2)^{3/2}\left(1/N_0^{1/2}\right)\left[\omega_{\mathbf{q}s}\omega_{\mathbf{q}_1s_1}\omega_{\mathbf{q}_2s_2}\right]^{-1/2} \\
&\quad\times \sum_{NP}\sum_{\mu\nu\pi}\sum_{\alpha\beta\gamma}\Psi_{\alpha\beta\gamma}\left(0\mu, N\nu, P\pi\right)\left(M_\mu M_\nu M_\pi\right)^{-1/2} \\
&\quad\times e^{i\mathbf{q}_1\cdot\mathbf{R}(N)} e^{i\mathbf{q}_2\cdot\mathbf{R}(P)} \\
&\quad\times w_\alpha\left(\mathbf{q}s,\mu\right) w_\beta\left(\mathbf{q}_1s_1,\nu\right) w_\gamma\left(\mathbf{q}_2s_2,\pi\right)
\end{aligned} \tag{5}$$

and,

$$\begin{aligned}
\Psi_{\lambda\lambda_1\lambda_2\lambda_3} &= \Psi_{\mathbf{q}s,\mathbf{q}_1s_1,\mathbf{q}_2s_2,\mathbf{q}_3s_3} \\
&= (\hbar/2)^2\left(1/N_0\right)\left[\omega_{\mathbf{q}s}\omega_{\mathbf{q}_1s_1}\omega_{\mathbf{q}_2s_2}\omega_{\mathbf{q}_3s_3}\right]^{-1/2} \\
&\quad\times \sum_{NPQ}\sum_{\mu\nu\pi\rho}\sum_{\alpha\beta\gamma\eta}\Psi_{\alpha\beta\gamma\eta}\left(0\mu, N\nu, P\pi, Q\rho\right)\left(M_\mu M_\nu M_\pi M_\rho\right)^{-1/2} \\
&\quad\times e^{i\mathbf{q}_1\cdot\mathbf{R}(N)} e^{i\mathbf{q}_2\cdot\mathbf{R}(P)} e^{i\mathbf{q}_3\cdot\mathbf{R}(Q)} \\
&\quad\times w_\alpha\left(\mathbf{q}s,\mu\right) w_\beta\left(\mathbf{q}_1s_1,\nu\right) w_\gamma\left(\mathbf{q}_2s_2,\pi\right) w_\eta\left(\mathbf{q}_3s_3,\rho\right)
\end{aligned} \tag{6}$$

respectively. Here, $\Psi_{\alpha\beta\gamma}$ and $\Psi_{\alpha\beta\gamma\eta}$ are the real-space third and fourth-order interatomic force constants (IFCs), α, β, γ and η are the Cartesian indices, N, P and Q are the indices for the lattice positions with lattice vectors $\mathbf{R}(N), \mathbf{R}(P)$ and $\mathbf{R}(Q)$ respectively, μ, ν, π and ρ are the indices for the basis atoms, $[\mathbf{w}(\mathbf{q}s, \mu)]_\alpha = w_\alpha(\mathbf{q}s, \mu)$ is the αth component of the eigenvector for the phonon mode $(\mathbf{q}s)$, M_μ is the mass of the μth basis atom, N_0 is the total number of $\mathbf{q}$-points in the Brillouin zone and $\hbar = h/2\pi$ with h being the Planck's constant. The phonon-isotope scattering probabilities $(W^{\text{iso}}_{\lambda\lambda_1})$ are given by [18],

$$W^{\text{iso}}_{\lambda\lambda_1} = \frac{\omega_\lambda^2}{4N_0} \sum_\sigma g'(\sigma) \left|\mathbf{w}(\sigma, \lambda) \cdot \mathbf{w}^*(\sigma, \lambda_1)\right|^2 \delta(\omega_\lambda - \omega_{\lambda_1}) \tag{7}$$

where $g'(\sigma) = \left(1/\bar{M}_\sigma^2\right) \sum_\zeta f_{\zeta\sigma} \left(M_{\zeta\sigma} - \bar{M}_\sigma\right)^2$ is a mass variance parameter with $f_{\zeta\sigma}$ and $M_{\zeta\sigma}$ being the concentration and mass of the ζth isotope of the σth atom respectively and $\bar{M}_\sigma$ is the average mass of the σth atom. The energy-conserving δ-functions in Supplementary Equations 5, 6 and 7 are computed using the analytical tetrahedron method [19].

The expression for the fourth-order anharmonic Helmholtz free energy $(F_{4^{\text{th}}-\text{order}})$, which is used to determine the pressure in our calculations, is given by: [12, 20]

$$\begin{aligned} F_{4^{\text{th}}-\text{order}} = \Psi_0 + \underbrace{\sum_{\mathbf{q}s} \left[\frac{1}{2}\hbar\omega_{\mathbf{q}s} + k_B T \log\left[1 - e^{-\hbar\omega_{\mathbf{q}s}/k_B T}\right]\right]}_{\text{F}_\text{H}} \\ + \underbrace{\frac{1}{2} \sum_{\mathbf{q}s\mathbf{q}'s'} \Psi_{\mathbf{q}s,-\mathbf{q}s,\mathbf{q}'s',-\mathbf{q}'s'} \left(n_{\mathbf{q}s} + \frac{1}{2}\right)\left(n_{\mathbf{q}'s'} + \frac{1}{2}\right)}_{\text{F}_4} \\ \overbrace{-\frac{1}{2\hbar} \sum_{\mathbf{q}\mathbf{q}'\mathbf{q}'} \sum_{ss's''} \left(\left|\Psi_{\mathbf{q}s\mathbf{q}'s'\mathbf{q}''s''}\right|^2 \left[\frac{n_{\mathbf{q}s}n_{\mathbf{q}'s'} + n_{\mathbf{q}s} + \frac{1}{3}}{(\omega_{\mathbf{q}s} + \omega_{\mathbf{q}'s'} + \omega_{\mathbf{q}''s''})_p} + \frac{(2n_{\mathbf{q}s}n_{\mathbf{q}''s''} - n_{\mathbf{q}s}n_{\mathbf{q}'s'} + n_{\mathbf{q}''s''})}{(\omega_{\mathbf{q}s} + \omega_{\mathbf{q}'s'} - \omega_{\mathbf{q}''s''})_p}\right] + 2\Psi_{\mathbf{q}s,-\mathbf{q}s,\mathbf{q}''s''}\Psi_{\mathbf{q}'s',-\mathbf{q}'s',-\mathbf{q}''s''} \frac{\left(n_{\mathbf{q}s}n_{\mathbf{q}'s'} + n_{\mathbf{q}s} + \frac{1}{4}\right)}{(\omega_{\mathbf{q}''s''})_p}\right)}^{\text{F}_3} \end{aligned} \tag{8}$$

where Ψ_0, F_H, F_3 and F_4 are the electronic, harmonic, third-order and fourth-order parts of the total anharmonic free energy.

[1] Datchi, F., Dewaele, A., Le Godec, Y. & Loubeyre, P. Equation of state of cubic boron nitride at high pressures and temperatures. *Phys. Rev. B* **75**, 214104 (2007).

[2] Knittle, E., Wentzcovitch, R. M., Jeanloz, R. & Cohen, M. L. Experimental and theoretical equation of state of cubic boron nitride. *Nature* **337**, 349–352 (1989).

[3] Solozhenko, V. L., Husermann, D., Mezouar, M. & Kunz, M. Equation of state of wurtzitic boron nitride to 66 GPa. *Appl. Phys. Lett.* **72**, 1691–1693 (1998).

[4] Speziale, S., Zha, C.-S., Duffy, T. S., Hemley, R. J. & Mao, H.-k. Quasi-hydrostatic compression of magnesium oxide to 52 GPa: Implications for the pressure-volume-temperature equation of state. *J. Geophys. Res. : Solid Earth* **106**, 515–528 (2001).

[5] Garrity, K. F., Bennett, J. W., Rabe, K. M. & Vanderbilt, D. Pseudopotentials for high-throughput DFT calculations. *Comput. Mater. Sci.* **81**, 446–452 (2014).

[6] Tian, F. *et al.* Unusual high thermal conductivity in boron arsenide bulk crystals. *Science* **361**, 582–585 (2018).

[7] Li, S. *et al.* High thermal conductivity in cubic boron arsenide crystals. *Science* **361**, 579–581 (2018).

[8] Kang, J. S., Li, M., Wu, H., Nguyen, H. & Hu, Y. Experimental observation of high thermal conductivity in boron arsenide. *Science* **361**, 575–578 (2018).

[9] Dames, C. Ultrahigh thermal conductivity confirmed in boron arsenide. *Science* **361**, 549–550 (2018).

[10] He, L. *et al.* Accuracy of generalized gradient approximation functionals for density-functional perturbation theory calculations. *Phys. Rev. B* **89**, 064305 (2014).

[11] Jain, A. & McGaughey, A. J. Effect of exchange-correlation on first-principles-driven lattice thermal conductivity predictions of crystalline silicon. *Comput. Mater. Sci.* **110**, 115–120 (2015).

[12] Ravichandran, N. K. & Broido, D. Unified first-principles theory of thermal properties of insulators. *Phys. Rev. B* **98**, 085205 (2018).
[13] Arrigoni, M. & Madsen, G. K. H. Comparing the performance of LDA and GGA functionals in predicting the lattice thermal conductivity of III-V semiconductor materials in the zincblende structure: The cases of AlAs and BAs. *Comput. Mater. Sci.* **156**, 354–360 (2019).
[14] Xia, Y. Revisiting lattice thermal transport in PbTe: The crucial role of quartic anharmonicity. *Appl. Phys. Lett.* **113**, 073901 (2018).
[15] Lindsay, L. & Broido, D. A. Three-phonon phase space and lattice thermal conductivity in semiconductors. *J. Phys. Condens. Matter.* **20**, 165209 (2008).
[16] Feng, T., Lindsay, L. & Ruan, X. Four-phonon scattering significantly reduces intrinsic thermal conductivity of solids. *Phys. Rev. B* **96**, 161201 (2017).
[17] Dalton, D. A., Hsieh, W.-P., Hohensee, G. T., Cahill, D. G. & Goncharov, A. F. Effect of mass disorder on the lattice thermal conductivity of MgO periclase under pressure. *Sci. Rep.* **3**, 2400 (2013).
[18] Tamura, S.-i. Isotope scattering of dispersive phonons in Ge. *Phys. Rev. B* **27**, 858–866 (1983).
[19] Lambin, P. & Vigneron, J. P. Computation of crystal green's functions in the complex-energy plane with the use of the analytical tetrahedron method. *Phys. Rev. B* **29**, 3430–3437 (1984).
[20] Wallace, D. C. *Thermodynamics of crystals* (Courier Corporation, 1998).
